# A short history of time use research; implications for public health

**DOI:** 10.1186/s12889-019-6760-y

**Published:** 2019-06-03

**Authors:** Adrian Bauman, Michael Bittman, Jonathan Gershuny

**Affiliations:** 1 Sydney School of Public Health, Sydney University, Level 6, Building D17, Sydney, NSW 2006 Australia; 2 University of New England, Armidale, NSW Australia; 3 Centre for Time Use Research, Nuffield College, Oxford University, Oxford, UK

**Keywords:** Physical activity, Time use, Public health, History

## Abstract

**Background:**

This section defined time use (TU) research, illustrating its relevance for public health. TUR in the health context is the study of health-enhancing and health-compromising behaviours that are assessed across a 24 h day. The central measurement is the use of Time Use Diaries, which capture 24–48 h, typically asking about behaviour in each 15-min period. TUR is used for understanding correlates of health behaviours, and as a form of population surveillance, assessing behavioural trends over time.

**Main body:**

This paper is a narrative review examining the history of time use research, and the potential uses of TU data for public health research. The history of TUR started in studies of the labour force and patterns of work in the late 19th and early twentieth century, but has more recently been applied to examining health issues. Initial studies had a more economic purpose but over recent decades, TU data have been used to describe the distribution and correlates of health-enhancing patterns of human time use. These studies require large multi-country population data sets, such as the harmonised Multinational Time Use Study hosted at the University of Oxford. TU data are used in physical activity research, as they provide information across the 24-h day, that can be examined as time spent sleeping, sitting/standing/light activity, and time spent in moderate-vigorous activities. TU data are also used for sleep research, examining eating and dietary patterns, exploring geographic distributions in time use behaviours, examining mental health and subjective wellbeing, and examining these data over time. The key methodological challenge has been the development of harmonised methods, so population TU data sets can be compared within and between-countries and over time.

**Conclusions:**

TUR provides new methods for examining public health research questions where a temporal dimension is important. These time use surveys have provided unique data over decades and in many countries that can be compared. They can be used for examining the effects of some large public health interventions or policies within and between countries.

## Background

### What is time use research?

Public health researchers are becoming interested in the behaviours and attributes that can be measured across the 24 h day, and in the interrelationships of health-enhancing and health-compromising behaviour across a temporal spectrum. This area of research, time use studies, has a long history, and started over a century before its health and behavioural relevance was ever imagined.

Everything we do, we do in time. Just as in physics and spatial navigation, so in social science and now in public health, the improved measurement of behaviours and activities is of increasing importance. The order, duration and characteristics of the various different sorts of daily activities (e.g. paid work, physical exercise, eating, sleeping) as well as their intensity and context determine beneficial health outcomes and adverse health consequences. Recall questionnaire approaches may be of limited use for these purposes, partly due to recall bias, and partly since respondents may be unaware of the total amounts of time devoted to specific activities [1, 2]. By contrast, Time Use diaries also allow researchers to assess if there has been any compensation for changes in activity elsewhere during the day. An appropriate measurement technique would be to collect uninterrupted sequential accounts of a continuous stream of activities, together with estimated start/finish times, throughout a specified observation period: a “time use diary” (TUD), from which researchers could estimate durations of each activity by category, and within specific contexts.

Time use diaries record continuous events and actions through a 24 or 48 h period, sometimes longer. Diarists self-report their actions at specified periods, often 15 min epochs, into a TUD that reflects and codes 24 h for that individual. Constructing such accounts is rendered problematical by recall failures, the likelihood of multiple concurrent activities (e.g. reading and eating), and of competing alternative descriptions of activities (e.g. “walking” vs “travel to work”). So optimal time use data would collect information of multiple descriptive characteristics of each successive “event”, where the “event” itself is defined as the period during which all of these characteristics remain unchanged. Originally TUDs contained a main activity field, several concurrent or simultaneous activity fields, a location field and multiple “others co-present” fields [3]. Current practice now often has a field about device use, sometimes adds one or more affect fields (estimating levels of enjoyment, stress or others), as well as fields containing measurements in real time taken from instruments (such as accelerometers or global position system [GPS] devices) carried by the diarist.

Until recently diary survey designers had to choose between either using “own words” to produce an extensive and difficult to code list of self-reported activities or less demanding “light-diary” activity-list studies, which were cheaper, but less useful insofar as the maximum practicable 40 or so listed activity categories were far fewer than the 300 or more distinct activities derived from the own-words design. Now, however, the use of computer-aided or internet-based interview techniques, provides the possibility of using “unfolding” (sequentially nested) fixed lists with levels of detail rivalling those of own-words surveys.

The aims of this paper are to describe the history and trends in time use surveys, and their application to public health research. A specific focus in the potential for time use research in understanding physical activity patterns and trends in populations.

## Main text

### The history of time use

There is a surprisingly long history of time-diary studies, with initial work focusing on describing social conditions, monitoring economic productivity and providing labour force information. It appears that the original innovators were Russian *zemstvo (rural county or region administrative unit)* researchers from before 1900, observing peasant households. The results however survive only in the form of calculations of the balance of different sorts of peasant work [4]. The British Fabian Socialist, Maud Pember-Reeve persuaded a small sample of London women to complete time use diaries, particularly to demonstrate food distributional inequalities related to poverty and high infant mortality [5]. Bevans’ 1913 Columbia University doctoral thesis on London factory workers, identified by Sorokin as a time-diary study [6], emerges from further inspection as deriving from a rather different methodology [7]. The Soviet Russian economist Stanislav Strumlin published a time budget account of Russian workers in 1925 [8]. And almost simultaneously the United States (US) Department of Agriculture (USDA) started to collect time diaries from farm, town, and later elite educated “college”, women [9], providing the earliest time diary dataset whose original individual-level data survives for use by modern researchers [10].

The Soviet interest in time allocations was driven by the idea of a Marxist central planned economy believing the worth or value of objects to be determined the necessary labour time it embodies. By the 1920s the Soviets had become attracted to the concept of Scientific Management, pioneered in the US and elsewhere by F.W. Taylor. He called his technique, using stop-watches to rationalize the process of production, ‘time studies’. The fact that the industrial revolution relied upon wage labour, bought and sold by units of time, underpinned any practices aimed at increasing productivity (greater output in a shorter time). State socialist societies expected increase production to expand human freedom, resulting a ‘leisure revolution’, an idea also popular in the West during the 1960s [11].

Time use research developed in the late 1920s in the US coincident with a larger narrative about the position of women in the early twentieth century and the influence of the Domestic Science movement. ‘Homemaking’, was gradually influenced by advances in hygiene and nutrition, which greatly increased life expectancy. With farm consolidation, the balance of women’s employment changed from live-in domestic service (wealthier households complained of a ‘servant crisis’) towards factory or office employment. In the mid-twentieth Century, US homes experienced a technological revolution, with electrification, washing machines and fridges, alongside upgraded plumbing (hot and cold running water and indoor bathrooms). The rise in domestic productivity changed ‘time use’ in unexpected ways. Clothes became ‘dirty’ after a single wearing, shopping volume increasing, and combined with increasingly elaborate child-raising practices, some US households experienced unexpected increases in unpaid domestic work time, a paradoxical consequence of ‘labour-saving’ devices. In the 1920s, an American economist (Hildegard Kneeland) extended the USDA time use research programme to include samples of “town women” and finally “college women” and published a pioneering time use estimates of the economic value of US women’s homemaking activity based on the USDA materials [9].

The first major US academic study of the time use of both men and women, *(*which explicitly identified Kneeland’s USDA work as the source of its methodology) was published by Lundberg and colleagues in 1934 [12]. Their study of leisure in a suburban community, was followed by Sorokin and Berger’s 1939 [6] investigation of the activity patterns of young people. The first time-diary-type research in the United Kingdom (UK) was conducted by the left-leaning Mass Observation organisation in 1937 (more than 1000 of the original diaries survive to the present), closely followed by the BBC Department of Audience Research which conducted its first “listener availability” study in 1938. This led to data collections estimating the daily habits of radio listeners, and later television viewers, and were used to guide appropriate programming and for assessing target market sizes for advertisers.

Robinson and Converse [13] studied historical change in US time use. Time studies comparing societies with varying rates of television set ownership suggest that television has had a greater impact on time allocation than the so-called information technology revolution at the end of twentieth century. Television displaced equivalent time spent listening to radio and going to movies but had smaller effects on sleep, out-of-home socializing and reading, with minor effects on home chores and grooming [14]. By the early 1960s large diary-based time use studies were underway in Czechoslovakia, France, Hungary, Poland, Japan the UK and many other countries.

This extensive pre-history means that by the time of the first properly designed, *ex-ante* (pre-fieldwork) harmonised cross-national time use study, funded in the mid-1960s by UNESCO and led by the Hungarian sociologist Alexander Szalai [3] there was already a considerable international convergence of research practice. The 12-country dataset that emerged from the Szalai study, one of the first cross-national comparative studies on any subject available to social scientists, popularised a design of time diary studies that continues, with minor variations, to the present time.

This common underlying design means that *ex-post* harmonisation of the micro-level data of all the subsequent time-diary based studies whose samples have survived to the present time is, requires post-hoc harmonisation using already collected data. When surveys do not have identical response categories, an alternative is to find a common denominator by collapsing categories. In relation to time use surveys, most national activity classification are derived from schema pioneered by the Szalai multinational study in 1965 [3], so this is a feasible task. And the next substantial exercise in *ex-ante* harmonised cross-national comparative time diary research, organised in the planning of TUD collections by Eurostat from the mid-1990s, started from the same Szalai-based model [15, 16]; the two tranches of national data (1999–2006 and 2009–2015) collected so far include all the larger European Union countries.

The US started to collect the American Time Use Survey as the seventh wave of the Current Population Survey in 2003. The objectives of this survey changed during the twentieth Century; the surveys were initially focused on patterns of time spent in work and family life, and using these surveys to assess one dimension of economic outputs. A particular focus was the changing time allocations for women at work and at home. Time use surveys captured all activities in a day and offered a window into the emerging problem of reconciling family responsibilities and seeking a career. The United States Department of Agriculture also financed a supplement on food-related matters. The American Time Use Survey departs from the basic Szalai architecture, by only collecting a single activity category per event. But it is collected continuously, with annual releases of data, and is the largest source of time diary data collected anywhere. Having a continuous collection opens the way for workforce analyses of how economic downturns affect health both directly (rates of morbidity and mortality rise) and indirectly through any changes to ‘lifestyle’ risk factors. An additional feature of TUDs is that domains of interest can be aggregated into different combinations. For example, time use components that might reflect economic productivity can be aggregated into ‘total working time’, but some of these same components may be differently combined with other parts of the day, for example, to estimate total daily sitting time. This inherent flexibility of TUD has led to its recent use in assessing health and lifestyle-related dimensions, including active time and sedentary time.

The Multinational Time Use Study (MTUS), maintained by the Centre for Time Use Research, University of Oxford, is by far the largest available collection of comparative and historical time use materials, with 1.2 M days from 85 surveys from 26 countries, all harmonised *ex post,* most of the data freely downloadable for use by academic researchers (http://www.timeuse.org/mtus) [17]. The recent year TUD samples for 25 countries are shown in Table 1.

**Table 1:** MTUS: N of diary days for historical cross-national comparative research on time use patterns

Country	1961–6	1971–5	1979–81	1983–7	1989–92	1995–99	1999–04	2005–09	2010–15	Country Totals
Austria					25,233					25,233
Australia		1491		3181	13,806	14,315		13,617		46,410
Belgium	2085									2085
Bulgaria	2096									2096
Canada		2138	8727	9618	25,233	10,726		19,597	15,390	91,429
Czech Rep	2211									2211
Germany	5078				25,812		35,813			66,703
Denmark	4174			3561			6617			14,352
Spain							51,813	19,295		71,108
Finland			12,038	15,184		10,074		7480		44,776
France	2791	4634				15,441		27,903		50,769
Hungary								8391		8391
Israel					4843					4843
Italy			2118		38,110		51,206	40,940		132,374
S Korea						85,906		40,526		126,432
Neth’lnd		9163	19,110	22,841	23,905	22,589	12,691	15,428		125,727
Norway		6516	6066		6129		7669			26,380
Peru	777									777
Poland	2740						40,292		76,656	119,688
Serbia	1993									1993
Sweden					7065		7727			14,792
Slovenia	2120						12,276			14,396
UK	9292	20,252		16,316			20,982		13,538	80,380
US	2017	7088		3339			34,693	64,085	38,182	149,404
S Africa							14,302			14,302
Year totals	37,374	51,282	48,059	74,040	170,136	159,051	296,081	257,262	143,766	1,237,051

*MTUS* multinational time use survey, *Czech Rep* Czech Republic, *S Korea* South Korea, *Neth’lnd* Netherlands, *UK* United Kingdom, *US* United States, *S Africa* South Africa

TUDs have been assessed for validity in relation to different criteria, and generally show good measurement properties. With respect to physical activity measurement, TUDs have been compared to objective accelerometry measures, and show better concordance than usual self-report physical activity questionnaires [2]. Correlations with accelerometry-assessed sedentary time are around 0.58, and range from 0.45–0,69 for moderate-vigorous physical activity, higher than the usual coefficients between self report and objectively assessed activity [2].

### Time use and its public health research potential

Time use surveys are a valuable resource for public health researchers (i) as they provide comprehensive coverage of all activities during the 24 h day and (ii) their long history, national representativeness and largely standardised form, facilitates epidemiological research into cross national comparisons and trend analyses over time. Time use data have been used to describe physical activity patterns, mental health states, and trends in nutrition that are relevant to public health. In addition, socio-economic inequalities in these health attributes, as well as more broadly in society, can also be gleaned from trend analyses. However, the range of potential applications has not been thoroughly explored in these public health contexts.

With respect to physical activity, TUDs capture the spectrum of activity, including moderate-vigorous time, through light intensity activity, to sedentary (sitting) time and time spent in sleep, summating to the 24 h day. This brings a comprehensive dimension to energy expenditure research, using comparable TUDs over decades, compared to frequent changes to physical activity self-report surveillance measures. Further, TUDs provide unique contextual and domain specificity for physical activity behaviours, attributes that are not easily measured in objective monitoring. Validation work around TUDs against objective measurement with accelerometry indicate that TUDs show higher validity coefficients than almost all other self-reported physical activity measures [2], and additionally, provide information about the social and environmental contexts in which activities occur. TUD information has been used to profile physical activity and sport participation patterns within countries [18–21] and over time [20, 22]. Efforts have commenced to harmonise TUD information against the physical activity compendia, better classifying activities by their energy expenditure [21].

Other aspects of physical activity can also be studied using time use data. The burgeoning of passive transport and car dependency has occurred at different rates across countries, which can be studied using time use data. Similarly, workplaces have become automated, with major increases in sedentary and light intensity activities at work over the past six decades [23]. Time use data also demonstrate the major contributions of walking to reaching recommended levels of physical activity [18]. Further, the effects of urban planning on car use, as opposed to walking, become obvious in examining time use surveys. A comparison of Australian households showed that people in households that did not have a car walked on average 15 min day, whereas those households with one car walked about 8 min a day, and those with 2+ cars walked 4 min a day or less. This allows the study of the effects of “automobilization” and of transport policies on physical activity levels over time and across countries.

Time use studies capture trends in average durations (and interruptions) in reported daily sleep time but this is only just beginning to be systematically investigated [24, 25]. Recent research interest in the trade-off between components across the physical activity spectrum, with an examination of temporal trade-offs, for example, between screen-time and sleep among adolescents.

This Special Issue also contains a trend analysis of what time use data reveals about changes in eating habits. Previous time use research has started to investigate dietary and eating patterns across and between societies. Patterns of eating out, compared to domestic food preparation, showed marked differences between European countries and Anglophone countries [UK and US; 26]. This line of research can identify cultural and country-level differences that may advance our understanding of the obesity epidemic, with more home-prepared food and less snacking behaviour in Europe than North America [26, 27]. In addition, spatial geographic applications used with time use data identified “food deserts” in the US, areas where distance travelled to healthy food supply and food shops was substantially greater for socially disadvantaged people [28].

Mental health, happiness and subjective wellbeing have also been assessed using time use methods [29]. This can be combined with new technologies, and assess patterns the variation in mood across the day, and compares between countries and over time, in a standardised and comparable manner. At the clinical end of mental health research, there is important information to be gained through an exploration of how people with serious mental illness spend their time during the day [30].

The future of 24-h daily measurement is likely to be a blend of TUD usage, alongside technological developments in several types of objective measures. Although accelerometers cannot measure activity type or context, newer developments in wrist-worn wearable trackers often provide reliable measurement, have inbuilt GPS assessment, and often can differentiate activity types [31]. They also provide 24-h measures and can assess sleep time and quality [32]. Other approaches to assessing behaviours across the day use random time sampling, and assessing instantaneous estimates of current behaviour; this method, ecological momentary analysis (EMA) has been applied to physical activity and sedentary behaviour research, mood assessment, and eating behaviours [33–35]. Extensions of this idea relate to smartphone and device apps and web interfaces for assessing time use more broadly [36]. Continuous front-facing wearable cameras {“sensecams”} have been tested, and algorithms are starting to be developed to validate physical activity and dietary intake across the waking day, and compared to TUD [37].

Physical activity researchers have become interested in the ‘spectrum of activity’ across the day, including sleep time, and 24 h guidelines are proposed in Canada; although these are based on continuous accelerometry data, they build on the time use concept, and are attempting to define the health thresholds for different dimensions of activity across 24 h [38]. Another variant derived from time use thinking is the concept of health-enhancing substitution of physical activity behaviours, for example, replacing sitting time with sleep time. This has generated new statistical techniques, ‘compositional data analysis’ (CoDA), which allow time use researchers to construct the day as a whole, rather than as separate activity domains, and model the effects on health outcomes of reallocating time from one domain to another [39].

## Conclusions

Maintaining TUDs is a worthwhile investment for health-related research, to maintain longterm cross-national, comparable trend information. Further, TUD also provides trend information on aspects of social inequalities, another fundamental precept of public health research. It may even be possible to compare the population effects longterm public policy interventions using TUDs, and hence use these surveys in the evaluation of some public health programs. Technology will complement self-report TUDs, and provide additional data and new ways of collecting time use information. These new methods may facilitate inexpensive larger scale data collections, but may also pose new challenges, such as maintaining representativeness in sampling, and preserving backwards comparability with the decades of TUDs using traditional survey methods. The use of TUD in low-middle income countries is relatively rare, but could be particularly useful in monitoring rapid trends in urbanisation and industrialisation that lead to increased sitting time and reduced total physical activity; this is a potential benefit of TUD in more countries, especially those undergoing epidemiological and demographic transition. Globally, TUD remains as a valuable, and under-utilised, public health data resource, which can help to explain the present, and track trends over many decades, in a way that is unique among any population measurements.

## Abbreviations

CoDA: Compositional data analysis
EMA: Ecological momentary analysis
GPS: global positioning system
MTUS: Multinational Time Use Study
TUD: time use diary
UK: United Kingdom
UNESCO: United Nations Educational, Scientific and Cultural Organisation
US: United States
USDA: United Stated Department of Agriculture
